# Perceived social support and characteristics of social networks of families with children with special healthcare needs following the COVID-19 pandemic

**DOI:** 10.3389/fpubh.2024.1322185

**Published:** 2024-02-29

**Authors:** Anne Geweniger, Michael Barth, Anneke Haddad, Henriette Högl, Shrabon Insan, Annette Mund, Thorsten Langer

**Affiliations:** ^1^Department of Neuropediatrics and Muscle Disease, Center for Pediatrics, Medical Center—University of Freiburg, Freiburg, Germany; ^2^Department of General Pediatrics, Adolescent Medicine and Neonatology, Center for Pediatrics, Medical Center—University of Freiburg, Freiburg, Germany; ^3^Kindernetzwerk e.V., Mainaschaff, Germany

**Keywords:** social support, support networks, children with special healthcare needs, caregivers, pandemic, Covid-19, children with chronic disease, inequalities

## Abstract

**Background:**

Children with special healthcare needs (CSHCN) require more support than the average of their peers. Support systems for CSHCN were particularly affected by pandemic control measures. Perceived social support is a resource for health and wellbeing for CSHCN and their families. Associations of social support, mental health and socioeconomic status (SES) have been described. This study aims to (1) assess perceived social support in families with and without CSHCN; (2) describe structure and types of social networks of families with and without CSHCN; and (3) explore associations between perceived social support, disease complexity, child and caregiver mental health, and SES.

**Methods:**

This is the third of a sequential series of cross-sectional online surveys conducted among caregivers of children ≤ 18 years in Germany since the beginning of the COVID-19 pandemic, administered between 1st December 2022 and 10 March 2023. The Brief Social Support Scale (BS6) assessed perceived social support. Child and parental mental health were assessed using the Strengths and Difficulties Questionnaire (SDQ) and WHO-5 Wellbeing index. The CSHCN-Screener identified CSHCN. Descriptive statistics and linear regression modeling assessed associations between perceived social support, parent-reported child mental health problems, disease complexity, caregiver mental wellbeing and SES.

**Results:**

The final sample included 381 participants, among them 76.6% (*n* = 292) CSHCN. 46.2% (*n* = 176) of caregivers reported moderate, i.e., at least occasional social support. Social support was largely provided by informal social networks consisting of partners, relatives and neighbors/friends. Linear regression modeling revealed associations of lower perceived social support with higher disease complexity of the child, lower caregiver mental wellbeing, lower SES and increasing caregiver age.

**Conclusion:**

The results of this study describe inequalities in perceived social support according to disease complexity of the child, caregiver mental health and socioeconomic status. They highlight the importance of social support and support networks as a resource for wellbeing of caregivers and CSHCN. Moving on from the COVID-19 pandemic, recovery strategies should focus on low-threshold interventions based in the community to improve social support for families with CSHCN and actively involve caregivers in identifying needs and co-creating new approaches.

## 1 Introduction

Children with special healthcare needs (CSHCN) have chronic health conditions which require more support than the average of their peers (1). Their families face multiple demands relating to the physical and mental wellbeing of the affected child, management of limited resources and to family functioning (2, 3). As a result, caregivers of CSHCN are more likely to be affected by low levels of mental wellbeing, increased levels of stress, financial difficulties, social isolation and difficulties in accessing community resources (2–4). A growing body of research highlights the complex and multi-faceted impact of the COVID-19 pandemic on families with CSHCN. Suspension or reduction in frequency of health care services provision led to an increase in care responsibilities with parents trying to maintain therapies and surveilling their child’s health status at home. Rehabilitation services for CSHCN are often school-based, and thus school closures affected these children’s access not only to education, but also to therapies crucial for their physical health. In addition, respite services as a source of short-term relief to families with CSHCN were mostly suspended (5–7). Social distancing measures led to a loss of family and social support networks. As a result, worsening caregiver and child mental health, increasing stress and financial insecurities due to job loss or part-time work have been reported (3, 4, 8–10).

The first case of COVID-19 in Germany was reported on 27 January 2020. The first national lockdown lasted from 22nd March until 4 May 2020, followed by periods of stronger restrictions and distancing measures such as nightly curfews in November 2020 to January 2021, April 2021, and December 2021 to February 2022. Schools closed completely from about the middle of March until 4 May 2020 and patterns of (partial) reopening mostly coincided with periods of easing pandemic control measures; precise dates of school closure varied slightly by federal state. All pandemic measures were lifted by February 2023 (11).

Social support is a widely acknowledged resource for health and wellbeing, and an important coping resource for families with CSHCN in particular. Social support can arise both from social contacts and social networks and perceived social support may be as important as actual support provided (2, 12). Different conceptualizations of social support exist. *Functional support* describes the extent to which relationships serve particular functions and provide resources. It can be further categorized as tangible support (e.g., practical help, financial support); emotional support (e.g., empathy, companionship); appraisal support (e.g., help in decision-making processes) and informational support (e.g., provision of advice or information relating to particular needs). *Structural support* describes size and types of social networks, frequency of contacts and existence of relationships (13–15).

To our knowledge, there has been limited research focusing on social support of families with CSHCN during the COVID-19 pandemic. A Brazilian study highlights the relevance of perceived social support for quality of life, caregiver burden and stress of caregivers of CSHCN, but no differences in perceived social support between families with and without CSHCN during the COVID-19 pandemic were found (16). In our previous two surveys among families with and without CSHCN in Germany during the COVID-19 pandemic we described associations of parent-reported child mental health problems with increasing disease complexity of the child, low caregiver mental wellbeing, low SES, and inadequate social support reported by caregivers (17, 18).

The importance of social support in this context is further emphasized by the potential long-term impact of the COVID-19 pandemic on CSHCN due to persisting unequal access to treatment (e.g., financial barriers in accessing telehealth), associated poor health outcomes such as developmental delays or delays in diagnosis and treatment, and dependence on multidisciplinary support. These indirect impacts of the pandemic in turn increase the vulnerability of an already particularly vulnerable group leading to calls that “inequities and prior disadvantage […] [be] addressed in current policies regarding the recovery of healthcare services” (19) (p. 18).

Based on the findings outlined above, the goal of our study in a phase of pandemic recovery is to examine dimensions of social support and support networks of families with and without CSHCN with a focus on implications for health and care service provision post COVID-19. In particular, this study aims to

1. Assess perceived social support in families with and without CSHCN.
2. Describe structure and types of social networks of families with and without CSHCN.
3. Explore associations between perceived social support, disease complexity, child and caregiver mental health, and socioeconomic status (SES).

## 2 Methods

### 2.1 Study design

This study is the third of a sequential series of cross-sectional online surveys since the onset of the pandemic: the first survey was conducted from August–October 2020 (18), the second from December 2020–March 2021 (17). This third survey was initiated in December 2022 when most pandemic restrictions and social distancing measures in Germany had been relaxed or abolished. It was administered via REDcap©, an online survey platform, between 1st December 2022 and 10 March 2023.

Caregivers of children ≤ 18 years who gave informed consent were included in the study. Participants were recruited through convenience and non-probabilistic snowball sampling, study promotion via partner organizations, social and public media, and through free access websites. Representatives of the Kindernetzwerk e.V., a large German patient organization for families with children with chronic disease and disabilities, were involved in the survey design, study promotion and disseminated study results to their members through newsletters and free access websites. The study is registered with the German Registry for Clinical Studies (DRKS00022868). Ethics approval was granted by the ethics committee of Freiburg University (Approval number 377/20).

### 2.2 Measures

#### 2.2.1 Brief Social Support Scale (BS6)

The Brief Social Support Scale (BS6) is a bi-factorial questionnaire assessing overall perceived social support as well as both emotional-informational and tangible support. It was developed based on the MOS Social support survey (20). Three items assess tangible and emotional-informational support, respectively, on a 4-point Likert Scale. A sum score for perceived social support ranging from 6 to 24 can be calculated as well as sum scores for each of the two subscales ranging from 3 to 12. The authors suggest a stratification of the overall score of perceived social support into low (6–11), moderate (12–17; at least occasional support), high (18–23; at least mostly supported) and very high (24; always supported). The BS6 was validated in a population-based sample of 15,010 participants in an existing German cohort study and showed good reliability with Cronbach’s alpha α = 0.86 for overall perceived social support (20). For the purpose of this study, the wording of the items on the tangible support scale was slightly adapted to be suitable to the situation of families with children.

#### 2.2.2 Social support networks

Drawing on an assessment of support networks for families in pediatric oncology included in the Psychosocial Assessment Tool (PAT) (21, 22), six items eliciting support networks for tangible, informational, appraisal and emotional support were developed. For each area of support, participants were asked who provided this kind of support. Multiple answers were possible. Response options included both informal support provided by partners, grandparents or relatives, neighbors or friends; and formal support provided by volunteers, family support services, home care services or others.

*Unmet support needs* were assessed by seven newly developed items which were created in a collaborative process together with representatives of the patient organization Kindernetzwerk e.V. Each item mentioned a potential area of unmet support, e.g., “Everyday tasks in the household” and participants were asked whether they agreed, disagreed or if the item did not apply.

#### 2.2.3 Socioeconomic status

As outlined in the National Health Interview and Examination Survey for Children and Adolescents (KiGGS) in Germany (23), an index measuring SES was constructed as the sum of three indicators: household net equivalent income, parental education and parental occupation. Household net equivalent income was calculated as the monthly net family income adjusted for household size using a modified scale proposed by the Organisation for Economic Cooperation and Development (OECD) (23). Weights were assigned to the household head (=1), any additional adult living in the same household (=0.5) and children (=0.3). The monthly net family income was divided by the sum of weights per household. For parental education and occupation, the respective higher level of each parent was assigned to each household. Each of the three dimensions of the SES index takes values of 1–7 and the final SES index ranges from 3 to 21, with lower values indicating a lower socioeconomic status.

#### 2.2.4 Children with special health care needs

The Children with Special Healthcare Needs Screener (CSHCN Screener) is a five-item parent-reported screening instrument which aims to identify children with chronic physical, mental, behavioral or other conditions who require more health and related services than the average of their peers (1). Higher scores indicate higher disease complexity and healthcare needs (24). We stratified children into three groups (25): no special healthcare needs (CSHCN score = 0), chronic conditions (CSHCN score ≤ 2) and complex chronic conditions (CSHCN score ≥ 3) (24).

#### 2.2.5 Strengths and Difficulties Questionnaire (SDQ)

The Strengths and Difficulties Questionnaire (SDQ) is an established and validated screening instrument for mental health problems in children and adolescents. It relates to child or adolescent behavior during the previous 6 months. The standard parent-reported version of the SDQ applies to children aged 4–16 years, with a preschool version differing in three items (26, 27). The Total Difficulties score covers four subscales (hyperactivity/inattention, emotional symptoms, conduct problems, peer problems) and ranges from 0 to 40, with higher scores indicating more serious mental health problems. Both the German standard parent-report version and the preschool version are valid and reliable instruments (28, 29). We used age-appropriate versions of the SDQ for caregivers of children older than 2 years and a cut-off of 13 or higher on the Total Difficulties Score (30, 31).

#### 2.2.6 WHO-5 Wellbeing Index

The WHO-5 Wellbeing Index (WHO-5) is a 5-question screening tool for mental health with good validity and reliability (32). The final score ranges between 0 and 100, with 100 representing the best imaginable mental wellbeing. The cut-off point for depression screening is 50 (32).

#### 2.2.7 Sociodemographic measures

Included age and gender, relationship status, education, occupation, monthly household income, household size, area of residence and country of birth. Caregiver education was categorized according to the international CASMIN classification (33).

### 2.3 Statistical methods

Participants with no more than three missing values in any of the following key variables were included in the analysis: BS6 total score, SDQ total score, WHO-5 total score, CSHCN Screener score and SES variables (monthly household income, occupation and education). Missing values for household net income (10.6%) were replaced by multiple imputation. Analyses involving the SDQ were restricted to children older than 2 years of age. Descriptive statistics comprised frequencies for social support network structures, comparisons of means for BS6 total score between families with and without CSHCN by independent t-tests and by Chi-Square test for the stratified BS6 total score. Homogeneity of variance was assessed by Levene’s Test for Equality of Variances. Simple linear regression modeling was performed for BS6 total score on CSHCN total score.

Multiple linear regression modeling was performed on complete datasets (*n* = 327; 86% of total sample size) to assess associations of perceived social support (BS6 total score) with disease complexity, child mental health, caregiver mental health and SES. Analyses were adjusted for age and gender. Sensitivity analyses were performed for tangible and emotional-informational support subscales, respectively. Multicollinearity between exposure variables was assessed by calculating the variance inflation factor (VIF). Analysis was performed using IBM SPSS Version 27.0.

## 3 Results

### 3.1 Sociodemographic characteristics

Of 478 persons accessing the survey, 425 fulfilled the inclusion criteria. Among these, 381 met the criteria for missing data in key variables as outlined above and were thus included in the final sample. Participants were mostly female, lived with their partner in the same household and had on average two children. Of all participants, 39.4% had already participated in the first and second round of this sequential survey. Further sociodemographic characteristics are displayed in Table 1.

**Table 1:** Sociodemographic characteristics (*N* = 381).

	Mean (SD)	Range
**Age in years**		
Responding parent *(N = 333)*	42.7 (6.4)	23–64
All children *(N = 355)*	9.3 (4.6)	1–18
Children with SHCN *(N = 275)*	9.6 (4.7)	1–18
Number of children per household	2.0 (0.9)	1–5
Household size (*N* = 342)	4.0 (1.1)	1–8
Household net equivalent income (*N = 342*; *monthly, in Euros*)	2,127 (856)	(486–6,190)
Partner living in the same household (*n* = 301)	2,228 (845)	(536–6,190)
Partner living in a different household (*n* = 9)	1,487 (533)	(852–2,277)
No partner (*n* = 29)	1,294 (482)	(486–2,692)

	*n*	%
**Gender of respondent *(N = 338)***		
Male	37	10.9
Female	301	89.1
**Gender of child (*N = 354*)**		
Male	179	50.6
Female	173	48.9
Diverse	2	0.6
**Relation to child *(N = 342)***		
Biological mother	290	84.8
Biological father	35	10.2
Other	17	5.0
**Relationship status *(N = 339)***		
With partner, in same household	301	88.8
With partner, not in same household	9	2.7
No partner	29	8.6
**Country of birth of parents *(N = 341)***		
Both in Germany	304	89.1
One parent in Germany, one elsewhere	28	8.2
Both elsewhere	9	2.6
**Place of residence *(N = 341)***		
City (>100,000 inhabitants)	137	40.2
Surroundings of a city with >100,000 inhabitants	26	7.6
Town (20,000–100,000 inhabitants)	59	17.3
Small town (5,000–20,000 inhabitants)	48	14.1
Rural municipality (<5,000 inhabitants)	71	20.8
**Disease complexity of child**		
No special healthcare needs (CSHCN = 0)	89	23.4
Chronic disease (CSHCN ≤ 2 criteria)	55	14.4
Complex chronic disease (CSHCN ≥ 3 criteria)	237	62
**Educational level according to CASMIN-Classification *(N = 341)***		
1a: Inadequately completed general education	0	
1b: General elementary education	4	1.2
1c: General elementary education and vocational qualification	8	2.3
2a: Intermediate general qualification and vocational qualification	70	20.5
2b: Intermediate general qualification	4	1.2
2c_gen: General maturity certificate	4	1.2
2c_voc: General maturity certificate and vocational qualification	76	22.3
3a: Lower tertiary education	37	10.9
3b: Higher tertiary education	141	41.3
**Employment status of respondent *(N = 339)***		
Inactive or unemployed	40	11.8
Short term or temporary employment	19	5.6
Part-time	208	61.4
Full-time	72	21.2
**Participation in previous rounds of the survey *(N = 335)***		
First survey August–October 2020	25	7.5
Second survey April–July 2021	43	12.8
Both first and second survey	132	39.4
Participation for the first time	135	40.3

Among all children, 76.6% (*n* = 292) had special healthcare needs. Of these, 78.8% (*n* = 230) had a physical impairment, 73.6% (*n* = 215) a behavioral or sensory impairment and 55.1% (*n* = 161) had impaired speech or understanding.

### 3.2 Perceived social support

The mean score for perceived social support was 13.4 (SD 4.1) for the total score, 5.7 (SD 2.2) and 7.8 (SD 2.6) for tangible and emotional-informational subscales, respectively. Stratification of the total score revealed that 46.2% (*n* = 176) of caregivers reported moderate, i.e., at least occasional social support (Table 2). There was strong evidence that caregivers of CSHCN (12.7; SD 3.8) perceived lower social support than caregivers without CSHCN (16.0; SD 3.8) (*t*[379] = 7.16, *p* < 0.001) with a difference of 3.3 points on the BS6 scale (95% CI 2.4; 4.2). When stratifying the total social support score, 44.5% (*n* = 130) caregivers of CSHCN reported low perceived social support compared to 9.0% (*n* = 8) caregivers of children without SHCN (χ^2^[df = 2] = 39.78, *p* < 0.001; Table 2).

**Table 2:** Perceived social support (Brief Social Support Scale BS6, *N* = 381).

	CSHCN		No CSHCN		Total	
	*n*	%	*n*	%	*n*	%
**Social support categories based on total score**						
Low	130	44.5	8	9.0	138	36.2
Moderate (at least occasional support)	122	41.8	54	60.7	176	46.2
High to very high (at least mostly supported to always supported)	40	13.7	27	30.3	67	17.6

Percentages displayed are column percentages. Chi^2^-Test: χ2(df = 2) = 39.78, *p* < 0.001.

Simple linear regression showed strong evidence for an association between perceived social support and disease complexity. BS6 total scores decreased with increasing CSHCN total score (Supplementary Table S1).

### 3.3 Support networks

**Figure 1:**
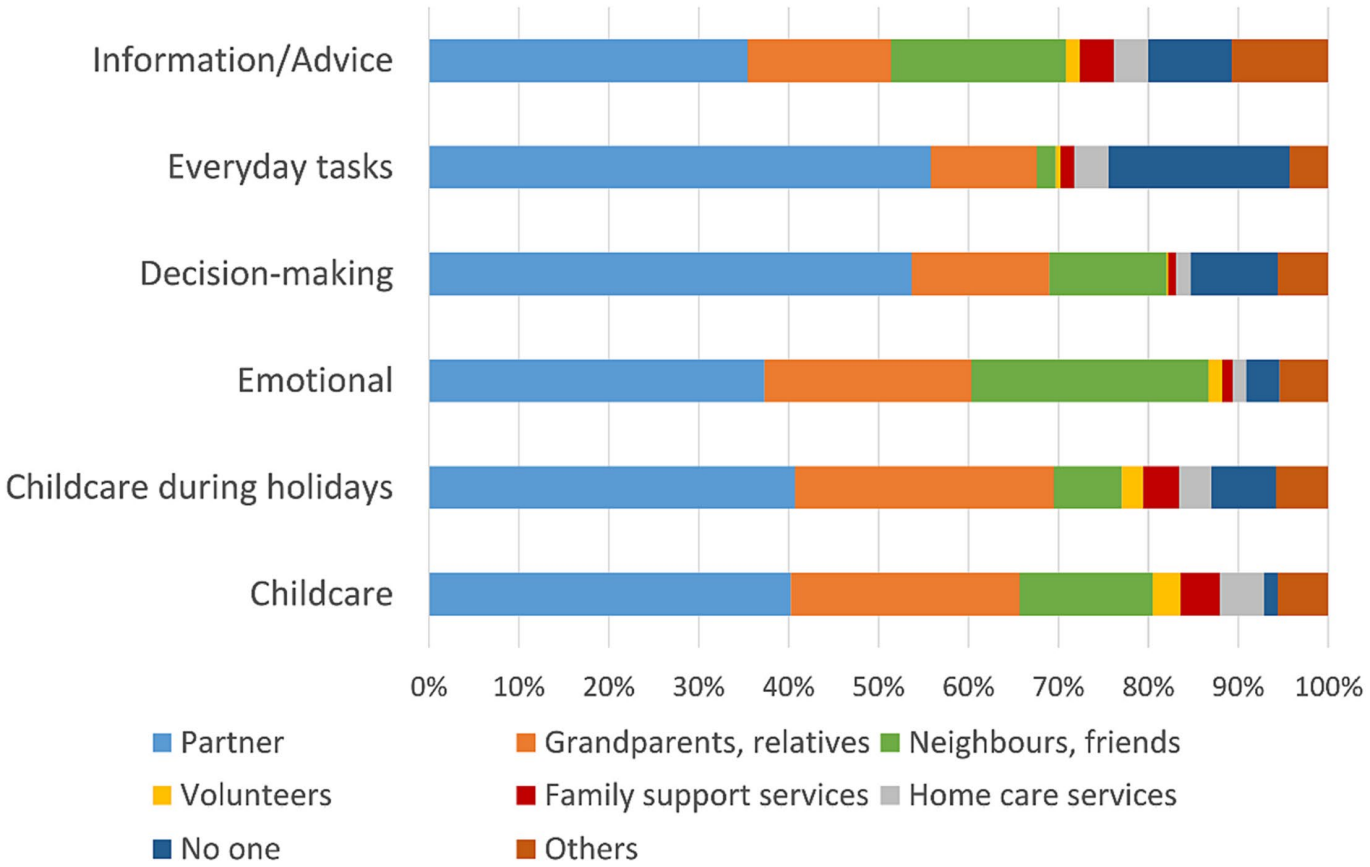
Support networks of families with CSHCN.

**Figure 2:**
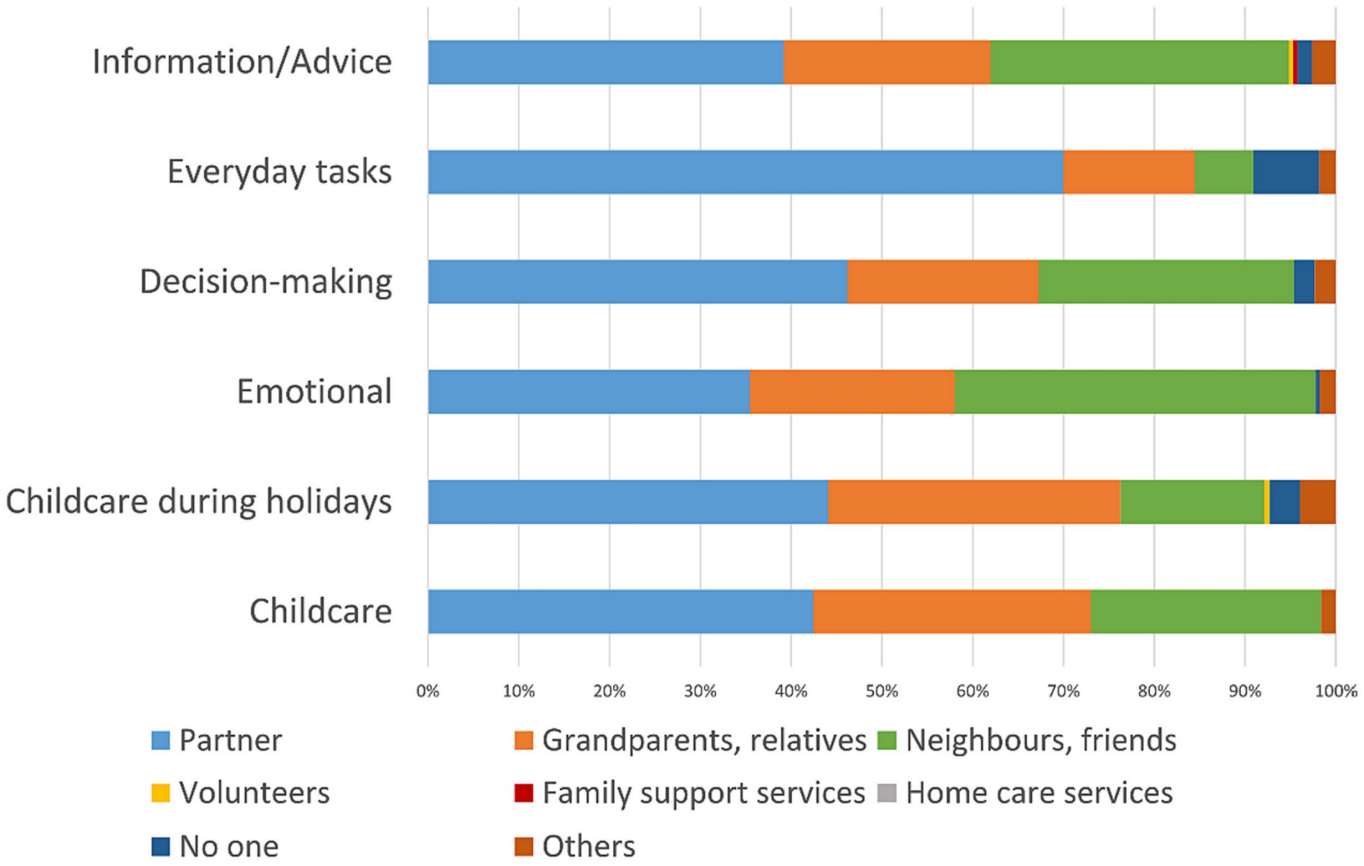
Support networks of families without CSHCN.

Results are displayed in Supplementary Table S2. Among all participants, support by a partner constituted the largest share of support provided for all items. In addition, grandparents or relatives mostly supported everyday childcare and childcare during holidays. Neighbors or friends were important sources of emotional and informational support or advice. Formal support provided by family support services or home care services only constituted a small share in the whole study population. Among families with CSHCN, family support services and home care services provided between 3.8% and 4.9% of support in childcare, everyday tasks and informational support compared to none for families without CSHCN (Figures 1, 2; Supplementary Tables S3, S4). However, support networks of both families with and without CSHCN were largely informal with support provided by a partner, grandparents or relatives and neighbors or friends.

Table 3 shows areas of unmet support needs of families with CSHCN. Support needs were highest for childcare outside school or nursery opening times (61%), everyday tasks in the household (60.1%) and support of the child in nursery or school (59.9%). Support needs were lowest for nursing or caring for a child with special healthcare needs, however this was still a relevant unmet need for more than one third of parents (36.4%). Stratified analysis revealed strong evidence for higher unmet needs among families with children with complex chronic disease compared to families with children with chronic disease for all items but financial support (results not displayed).

**Table 3:** Areas of unmet support needs of families with CSHCN.

	*n*	%	Total
Childcare outside nursery/school opening times	147	61	241
Support for the child in nursery/school	145	59.9	242
Supervision or support for siblings	84	42.4	198
Nursing/caring for the child with SHCN	88	36.4	242
Attending medical or therapy appointments	117	44.0	266
Everyday tasks in the household	161	60.1	268
Financial	112	44.6	251

### 3.4 Associations of perceived social support, disease complexity, child and caregiver mental health, and socioeconomic status

Results of the multiple linear regression modeling are displayed in Table 4. There was strong evidence of an association of perceived social support as measured by the BS6 total score, disease complexity, caregiver mental health, SES and age of caregiver. Perceived social support decreased with increasing disease complexity (CSHCN total score), decreasing caregiver mental wellbeing (WHO-5 score), decreasing SES and increasing caregiver age. After controlling for confounding effects of age, gender and disease complexity, there was no evidence of an association of perceived social support and parent-reported child mental health problems as measured by the SDQ total score. Overall, the model explained 22% of variance in perceived social support.

**Table 4:** Multiple linear regression modeling of BS6 total score on CSHCN total score, SDQ, WHO-5 and SES-Index (*N* = 327).

	Coefficient	SE	*t*	*p*	95%CI
Constant	17.34	2.06	8.44	<0.001	13.30; 21.39
CSHCN total score	**−0.44**	**0.13**	**−3.53**	**<0.001**	**−0.68; −0.19**
SDQ total score	−0.06	0.04	−1.64	0.103	−0.14; 0.01
WHO-5 total score	**0.04**	**0.01**	**3.38**	**<0.001**	**0.01; 0.06**
SES-Index	**0.21**	**0.07**	**2.82**	**0.005**	**0.06; 0.35**
Age of caregiver	**−0.13**	**0.04**	**−3.15**	**0.002**	**−0.22; −0.05**
Age of child	0.01	0.06	0.16	0.87	−0.11; 0.13
Gender of child	−0.71	0.41	−1.73	0.09	−1.51; 0.10
		**F**	**df**	***p***	**R** ^**2**^
		**13.06**	**7**	**<0.001**	**0.22**

BS6, Brief Social Support Scale; CSHCN, Children with Special Health Care Needs Screener; SES-Index, Index of socioeconomic status; WHO-5, WHO-5 Wellbeing Index, SDQ, Strengths and Difficulties Questionnaire. Analysis restricted to children aged > 2 years. Values for *p* ≤ 0.05 in bold.

Sensitivity analyses were performed for perceived tangible and emotional-informational support, respectively (Table 5). For perceived tangible support, there was strong evidence of an association with disease complexity, SES and caregiver age. Regarding perceived emotional-informational support, there was strong evidence for an association with disease complexity, caregiver mental health and caregiver age.

**Table 5:** Multiple linear regression modeling for tangible and emotional-informational support subscales (*N* = 327).

Tangible support	Coefficient	SE	*t*	*p*	95%CI
Constant	8.13	1.18	6.90	<0.001	5.81; 10.44
CSHCN total score	**−0.22**	**0.07**	**−3.10**	**0.002**	**−0.36; −0.08**
SDQ total score	−0.03	0.02	−1.17	0.242	−0.07; 0.02
WHO-5 total score	<0.001	0.006	−0.05	0.960	−0.01; 0.01
SES-Index	**0.12**	**0.04**	**2.95**	**0.003**	**0.04; 0.21**
Age of caregiver	**−0.07**	**0.02**	**−2.94**	**0.004**	**−0.12; −0.02**
Age of child	0.01	0.04	0.41	0.68	−0.06; 0.08
Gender of child	−0.37	0.23	−1.60	0.11	−0.06; 0.08
		**F**	**df**	***p***	**R** ^**2**^
		**7.13**	**7**	**0.001**	**0.14**
Emotional-informational support					
Constant	9.22	1.32	7.0	<0.001	6.62; 11.81
CSHCN total score	**−0.22**	**0.08**	**−2.73**	**0.007**	**−0.38; −0.06**
SDQ total score	−0.04	0.02	−1.50	0.134	−0.08; 0.01
WHO-5 total score	**0.04**	**0.007**	**5.31**	**<0.001**	**0.02; 0.05**
SES-Index	0.08	0.05	1.76	0.08	−0.01; 0.18
Age of caregiver	**−0.06**	**0.03**	**−2.28**	**0.02**	**−0.12; −0.01**
Age of child	−0.005	0.04	−0.12	0.91	−0.08; 0.07
Gender of child	−0.33	0.16	−1.27	0.21	−0.85; 0.18
		**F**	**df**	***p***	**R** ^**2**^
		**12.73**	**7**	**<0.001**	**0.22**

CSHCN, Children with Special Health Care Needs Screener; SES-Index, Index of socioeconomic status; WHO-5, WHO-5 Wellbeing Index, SDQ, Strengths and Difficulties Questionnaire. Analysis restricted to children aged > 2 years. Values for *p* ≤ 0.05 in bold.

There was no evidence for multicollinearity between independent variables included in the regression modeling.

## 4 Discussion

This study reports low to moderate levels of perceived social support in a sample of 381 families with and without CSHCN in Germany following the COVID-19 pandemic. Lower perceived social support was associated with higher disease complexity of the child, lower caregiver mental wellbeing, lower SES and increasing caregiver age. Social support was largely provided by informal social networks consisting of partners, relatives and neighbors or friends.

Perceived social support was lower in caregivers of CSHCN and associated with disease complexity of the child. Families of CSHCN face multiple responsibilities related to their child’s complex medical and psychosocial needs, and particularly rely on broad support networks (2). Studies conducted during the COVID-19 pandemic report a disintegration of family, peer and community support networks of caregivers of CSHCN (10, 34, 35). Caregivers of children with complex chronic disease were additionally affected by a lack of respite often provided through these networks (4, 9, 10, 35). However, a Brazilian study conducted during the first year of the pandemic did not find a difference in perceived social support between caregivers of children with and without developmental disabilities. The authors concluded that this was most likely due to social support being less available for everyone as pandemic restrictions affected all families (16). Barriers in accessing community support for families with CSHCN have been described prior to the COVID-19 pandemic (3). It remains open whether our results still reflect the impact of the pandemic years on perceived social support of caregivers of CSHCN and future studies are thus needed as we move beyond pandemic recovery.

In addition to the association of perceived social support and disease complexity, our study demonstrates that perceived social support decreased with lower caregiver mental wellbeing and lower SES. Financial stress and low SES have been described as associated with lower levels of perceived social support in adult populations (12, 20). Families with CSHCN are particularly at risk of financial difficulties due to part-time work and resulting income loss, and an association of chronic disease and disability with low SES has been widely described (3, 18, 36). It is crucial that efforts to strengthen social support focus on this vulnerable group and aim to remove barriers to accessing support systems.

Higher levels of psychological distress and mental health problems during the pandemic compared to pre-pandemic data have been reported for mothers in particular (17, 18, 37–39). Higher levels of depression in caregivers of CSHCN during the COVID-19 pandemic have been described for younger age, those being single or living alone, which might in addition point toward the importance of social support for caregiver mental wellbeing (40). However, our results indicated that decreasing perceived social support was associated not only with decreasing caregiver mental wellbeing but also with increasing caregiver age. This is contrary to results reported in a general population sample showing no relationship between perceived social support and age (20). Our finding may suggest that younger caregivers were better able to access social support during the COVID-19 pandemic, which warrants further exploration.

For families with and without CSHCN, social support constitutes a resource for lowering caregiver’s psychological distress and higher levels of emotional support showed positive effects on caregiver wellbeing (41). According to Wade et al., caregiver wellbeing is the central element in a family stress model and positively impacts children via changes in family processes, structure and organization (39). The most recent results of the representative German longitudinal COPSY study on youth mental health during the pandemic similarly describes a 4–14 times higher chance of better mental health outcomes in children with high social and family support (42). Accordingly, strengthening social support for families with and without CSHCN is an important mechanism for achieving both caregiver and child wellbeing.

In our study population, families largely relied on informal support networks. For families with CSHCN this might still be a reflection on reduced access to formal support services during the pandemic (8). However, those results highlight the importance of strengthening informal social support networks and increasing the availability of low-threshold support systems. Peer support interventions have the potential to act as egalitarian interventions without a power imbalance of the kind that exists, for example, between a formal service provider and the recipient. A recent Cochrane review on peer interventions for parents and carers of children with complex needs by Sartore et al. did not find clear evidence of an effect of the interventions on caregiver outcomes (2). However, this was mostly due to poor quality and heterogeneity of available studies. The authors still concluded that peer support might be equally effective as more intensive, standard interventions such as psychoeducation and stress management. Community health approaches such as neighborhood support programs can support families with CSHCN in everyday household tasks, attending medical appointments or providing childcare after school. Given that these programs are a valuable resource, patient organizations in Germany demand that they be strengthened (43). Further promising approaches include family guides for accessing community based social support and care coordination to enhance integration of medical and community-based supports for CSHCN (44, 45).

## 5 Limitations

The results of this study are limited by its design and recruitment process. The cross-sectional design does not allow inference of causality in the associations between social support, disease complexity, mental health and SES. Furthermore, the non-representative nature of the sample limits the generalizability of our study results. The recruitment process is likely to have encouraged a self-selection of participants, resulting in a sample with a high educational level. Participants from lower educational and occupational levels, those from a minority or ethnic background and families without CSHCN are underrepresented. Also, the survey delivery online might have excluded those from a low SES who lacked appropriate technology to access the survey. Associations described between perceived social support and low SES might thus still be underestimated. Similarly, differences in perceived social support between families with and without CSHCN might be either over- or underestimated.

## 6 Conclusion

The results presented here highlight the importance of social support and support networks as a resource for wellbeing of caregivers and children with special healthcare needs. Following the COVID-19 pandemic, we describe marked inequalities in perceived social support according to disease complexity, caregiver mental health and socioeconomic status. Recovery strategies and healthcare reform should focus on low-threshold interventions based in the community to improve social support for families with CSHCN, and actively involve caregivers in identifying needs and co-creating new approaches.

## Data availability statement

The raw data supporting the conclusions of this article will be made available by the authors, without undue reservation.

## Ethics statement

The studies involving humans were approved by Ethics Committee of Freiburg University (Approval number 377/20). The studies were conducted in accordance with the local legislation and institutional requirements. Written informed consent for participation in this study was provided by the participants’ legal guardians/next of kin.

## Author contributions

AG: Conceptualization, Data curation, Formal analysis, Investigation, Methodology, Project administration, Writing – original draft, Writing – review & editing. MB: Conceptualization, Writing – review & editing. AH: Conceptualization, Methodology, Supervision, Writing – review & editing. HH: Resources, Writing – review & editing. SI: Data curation, Writing – review & editing. AM: Resources, Writing – review & editing. TL: Conceptualization, Funding acquisition, Methodology, Project administration, Supervision, Writing – review & editing.
