# Supplementary material for: Perceived social support and characteristics of social networks of families with children with special healthcare needs following the COVID-19 pandemic
Source: Front Public Health. 2024 Feb 29;12:1322185. doi: 10.3389/fpubh.2024.1322185 (PMC10937572; doi:10.3389/fpubh.2024.1322185)
Supplement: Supplementary file 1 [file Table_1.DOCX]

Perceived social support and characteristics of social networks of families with children with special healthcare needs following the Covid-19 pandemic

Supplementary Material

**Table S1: Associations between social support and special healthcare needs (N=381).** Estimation by linear regression of BS6 total score on CSHCN total score. Estimates for regression coefficients are reported with their corresponding t-statistic, p-value and 95% confidence interval (**CI**). **BS6**: Brief Social Support Scale; **CSHCN:** Children with Special Health Care Needs.

|  | **Coefficient** | **SE** | **t** | **p** | **95%CI** |
| --- | --- | --- | --- | --- | --- |
| **BS6 total score** |  |  |  |  |  |
| CSHCN total score | -0.75 | 0.10 | -7.38 | <0.001 | -0.95; -0.55 |
| constant | 15.59 | 0.35 | 44.43 | <0.001 | 14.90; 16.28 |

**Table S2: Characteristics of social support networks (N=381).** All participants. Percentages given are percentages of total answers.

| **Support** | **Partner** | **Grandparents, relatives** | **Neighbours, friends** | **Volunteers** | **Family support services** | **Home care services** | **No one** | **Others** | **Total N answers** |
| --- | --- | --- | --- | --- | --- | --- | --- | --- | --- |
| Childcare | 40.8 | 26.6 | 17.4 | 2.4 | 3.4 | 3.8 | 1.1 | 4.6 | 800 |
| Childcare during school/nursery holidays | 41.8 | 29.8 | 9.7 | 1.9 | 3.0 | 2.7 | 5.8 | 5.3 | 677 |
| Emotional | 37.7 | 23.4 | 28.4 | 1.1 | 0.9 | 1.1 | 2.9 | 4.5 | 802 |
| Decision-making | 51.6 | 16.9 | 17.2 | 0.2 | 0.7 | 1.2 | 7.6 | 4.6 | 603 |
| Everyday tasks | 59.0 | 12.4 | 3.1 | 0.4 | 1.2 | 2.9 | 17.2 | 3.7 | 483 |
| Information/Advice | 36.5 | 17.9 | 23.1 | 1.3 | 2.7 | 2.9 | 7.2 | 8.5 | 694 |

**Table S3: Characteristics of social support networks of families with CSHCN.** Percentages given are percentages of total answers.

| **Support** | **Partner** | **Grandparents, relatives** | **Neighbours, friends** | **Volunteers** | **Family support services** | **Home care services** | **No one** | **Others** | **Total N answers** |
| --- | --- | --- | --- | --- | --- | --- | --- | --- | --- |
| Childcare | 40.2 | 25.4 | 14.8 | 3.1 | 4.4 | 4.9 | 1.5 | 5.6 | 607 |
| Childcare during school/nursery holidays | 40.8 | 28.8 | 7.6 | 2.4 | 4.0 | 3.6 | 7.2 | 5.8 | 503 |
| Emotional | 37.3 | 23.0 | 26.4 | 1.5 | 1.2 | 1.5 | 3.7 | 5.4 | 595 |
| Decision-making | 53.7 | 15.3 | 13.0 | 0.2 | 0.9 | 1.6 | 9.7 | 5.6 | 432 |
| Everyday tasks | 55.8 | 11.8 | 2.1 | 0.5 | 1.6 | 3.8 | 20.1 | 4.3 | 373 |
| Information/Advice | 35.4 | 16.0 | 19.4 | 1.6 | 3.8 | 3.8 | 9.3 | 10.7 | 505 |

**Table S4: Characteristics of social support networks of families without children with SHCN.** Percentages given are percentages of total answers.

| **Support** | **Partner** | **Grandparents, relatives** | **Neighbours, friends** | **Volunteers** | **Family support services** | **Home care services** | **No one** | **Others** | **Total N answers** |
| --- | --- | --- | --- | --- | --- | --- | --- | --- | --- |
| Childcare | 42.5 | 30.6 | 25.4 | 0 | 0 | 0 | 0 | 1.6 | 193 |
| Childcare during school/nursery holidays | 44.8 | 32.8 | 16.1 | 0.6 | 0 | 0 | 3.4 | 4.0 | 174 |
| Emotional | 38.6 | 24.6 | 34.3 | 0 | 0 | 0 | 0.5 | 1.9 | 207 |
| Decision-making | 46.2 | 21.1 | 28.1 | 0 | 0 | 0 | 2.3 | 2.3 | 171 |
| Everyday tasks | 70.0 | 14.5 | 6.4 | 0 | 0 | 0 | 7.3 | 1.8 | 110 |
| Information/Advice | 39.2 | 22.8 | 32.8 | 0.5 | 0.5 | 0 | 1.6 | 2.6 | 189 |
